# Intervertebral disc herniation effects on multifidus muscle composition and resident stem cell populations

**DOI:** 10.1002/jsp2.1091

**Published:** 2020-05-06

**Authors:** Obiajulu Agha, Andreas Mueller‐Immergluck, Mengyao Liu, He Zhang, Alekos A. Theologis, Aaron Clark, Hubert T. Kim, Xuhui Liu, Brian T. Feeley, Jeannie F. Bailey

**Affiliations:** ^1^ Department of Orthopaedic Surgery University of California San Francisco California USA; ^2^ Department of Orthopaedic Surgery San Francisco Veterans Affair Health Care System San Francisco California USA; ^3^ Department of Exercise Physiology Beijing Sport University Beijing China; ^4^ Department of Neurosurgery University of California San Francisco California USA

**Keywords:** paraspinal muscle, multifidus, satellite cells, fibro‐adipogenic progenitors, fatty infiltration, fibrosis, atrophy

## Abstract

**Background:**

Paraspinal muscles are crucial for vertebral stabilization and movement. These muscles are prone to develop fatty infiltration (FI), fibrosis, and atrophy in many spine conditions. Fibro‐adipogenic progenitors (FAPs), a resident muscle stem cell population, are the main contributors of muscle fibrosis and FI. FAPs are involved in a complex interplay with satellite cells (SCs), the primary myogenic progenitor cells within muscle. Little is known about the stem cell composition of the multifidus. The aim of this study is to examine FAPs and SCs in the multifidus in disc herniation patients. Multifidus muscle samples were collected from 10 patients undergoing decompressive spine surgery for lumbar disc herniation. Hamstring muscle was collected from four patients undergoing hamstring autograft ACL reconstruction as an appendicular control. Multifidus tissue was analyzed for FI and fibrosis using Oil‐Red‐O and Masson's trichrome staining. FAPs and SCs were visualized using immunostaining and quantified with fluorescence‐activated cell sorting (FACS) sorting. Gene expression of these cells from the multifidus were analyzed with reverse transcription‐polymerase chain reaction and compared to those from hamstring muscle. FI and fibrosis accounted for 14.2%± 7.4% and 14.8%±4.2% of multifidus muscle, respectively. The multifidus contained more FAPs (11.7%±1.9% vs 1.4%±0.2%; *P*<.001) and more SCs (3.4%±1.6% vs 0.08%±0.02%; *P*=.002) than the hamstring. FAPs had greater α Smooth Muscle Actin (αSMA) and adipogenic gene expression than FAPs from the hamstring. SCs from the multifidus displayed upregulated expression of stem, proliferation, and differentiation genes.

**Conclusion:**

The multifidus in patients with disc herniation contains large percentages of FAPs and SCs with different gene expression profiles compared to those in the hamstring. These results may help explain the tendency for the multifidus to atrophy and form FI and fibrosis as well as elucidate potential approaches for mitigating these degenerative changes by leveraging these muscle stem cell populations.

## Graphical abstract

Paraspinal muscles are known to develop muscle fatty infiltration (FI), fibrosis, and atrophy in a number of spine conditions, including intervertebral disc herniation. In this study, we histologically examined multifidus muscle specimens harvested from patients undergoing spine surgery for the degree of degenerative muscle pathology and characterized fibro‐adipogenic progenitors (FAPs) and Satellite cells (SCs) within the muscle in terms of quantity and gene expression profiles. We discovered a high degree of FI and fibrosis in the multifidus as well as large quantities of FAPs and SCs that displayed different gene expression profiles than those taken from healthy hamstring muscle controls.

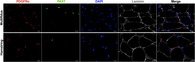

## 1 INTRODUCTION

Chronic low back pain (CLBP) is one of the world's leading debilitating conditions, and is the most common, noncancer reason for opioid prescription in the United States.^1^ The paraspinal muscles, particularly the *multifidus*, have an important biomechanical role by serving to stabilize posture and limit excessive intervertebral movement.^2^, ^3^ Clinically, paraspinal muscles have been shown to develop fatty infiltration (FI), fibrosis, and muscle atrophy in a number of spine conditions, such as scoliosis, spinal stenosis, and disc herniation.^4^, ^5^, ^6^, ^7^, ^8^ These degenerative changes are not without consequence as studies have found correlations between FI, muscle atrophy, back pain, and compensatory spinal biomechanics.^6^, ^9^, ^10^, ^11^, ^12^ The often multifaceted etiology of back pain creates ambiguity in understanding the pathophysiology underlying multifidus degeneration and symptoms across different spinal disorders, leaving room for further investigation.^4^, ^13^, ^14^

A better understood source of back pain is intervertebral disc herniation which often results in spinal nerve root compression, leading to denervation and associated multifidus FI and atrophy.^15^, ^16^, ^17^, ^18^ Changes in paraspinal histologic fibrosis profiles in patients with disc herniation have also been reported.^19^ In addition, avoidance of activities that worsen pain stemming from the herniation may lead to disuse related alterations that exacerbate these degenerative features.^20^, ^21^ As of now, strategies to mitigate FI and fibrosis in disc herniation patients are lacking, and attempts to reverse their progression in paraspinal muscle have proven to be challenging. For example, studies examining the effects of high intensity exercise regimens on muscle morphology in patients with CLBP have shown little or no efficacy in reducing paraspinal FI.^22^, ^23^ These results suggest the need to explore additional avenues to more effectively treat these patients.

Fibro‐adipogenic progenitors (FAPs), a subset of resident muscle stem cells characterized by platelet‐derived growth factor receptor alpha (PDGFRα) expression, have recently been found to serve an important role in muscle regeneration, FI, and fibrosis. Upon injury, FAPs rapidly proliferate and contribute to prodifferentiation signaling for satellite cells (SCs), the main myogenic progenitors in muscle. These signals increase the rate of new SC myotube formation and initially aid in overall muscle regeneration.^24^, ^25^, ^26^ However, after FAPs rapid expansion and transient promyogenic function, these cells continue toward adipocyte and fibroblast differentiation, becoming the main contributors of muscle FI and fibrosis.^27^, ^28^ Currently, little is known about the composition of paraspinal muscle in regard to FAPs and SCs and their association with paraspinal degeneration. A deeper understanding of such may provide opportunities for novel therapeutics that leverage these resident stem cell populations. In this study, we analyze multifidus muscle collected from disc herniation patients, examine the degree of degenerative pathology present, and investigate FAP and SC quantity and gene expression profiles. We hypothesized that there would be increased numbers of FAPs with greater adipogenic and fibrogenic expression profiles in the multifidus compared to control hamstring muscle.

## 2 METHODS

### 2.1 Muscle tissue collection

The need for institutional review board (IRB) approval for this study was considered prior to commencement. To access the vertebra for decompression surgery, muscle tissue, including the multifidus is dissected away from the spine for proper surgical exposure. Much of the muscle tissue adjacent to the facet joint is discarded during this process as gaining proper exposure results in significant disruption of the muscle‐bone interface which is not amenable to repair. Given that the collected muscle specimens were taken from tissue routed to surgical waste as part of the surgical approach, IRB approval was not required by our institution. Although IRB approval was not required from our institution, all muscle samples were properly deidentified to protect patient health information. Ten patients undergoing decompressive spine surgery for lumbar disc herniation were selected for multifidus waste collection. During the operation, small pieces of multifidus muscle removed from the lumbar region as surgical waste were collected. The samples were obtained from the muscle directly adjacent to and overlying the facet joint capsule that was removed as part of the surgical approach. The muscle removed from this location is reliably multifidus based on the anatomy and consistent surgical approach used by the surgeons. The tissue was then immediately transferred to the lab on ice for same day processing. Each specimen weighed approximately 1 g. The muscle samples were then partitioned for histology and fluorescence‐activated cell sorting (FACS) cell sorting. To serve as a control from an appendicular anatomic location, hamstring muscle was harvested from patients undergoing ACL reconstruction with hamstring tendon autograft.

### 2.2 Histology and immunofluorescence

Muscle specimens reserved for histology were snap frozen in liquid nitrogen‐cooled isopentane and underwent serially sectioning at −20°C at a thickness of 10 μm using a cryostat. Oil‐Red‐O(Sigma) and Masson's trichrome (American Mastertech) staining were used to identify fat and fibrosis, respectively. Sections used for immunostaining were fixed in 4% paraformaldehyde for 10 minutes, rinsed in phosphate buffered saline (PBS) for 10 minutes three times, placed in 10 mM sodium citrate solution at 95°C for 10 minutes, and washed again three times for 10 minutes in PBS. They were then covered with blocking solution (0.2% Triton X‐100, 2% bovine serum albumin in PBS) for 1 hour at room temperature. Primary antibodies directed against Laminin (rat/IgG, diluted 1:200, Abcam11576), PDGFRα (rabbit/IgG, 1:500, Invitrogen PA5‐16571) to identify FAPs, and Pax7 (mouse/IgG, diluted 1:50, Developmental Studies Hybridoma Bank) to identify SCs were diluted in a block mix and added to the sections for overnight incubation at 4°C. The next day, samples were rinsed in PBS three times for 10 minutes and then incubated in a mixture of the donkey anti‐rat Alexa Fluor 647 (Abcam, ab150155, 1:200), donkey anti‐rabbit Alexa Fluor 594 (Abcam, ab150108, 1:200), donkey anti‐mouse Alexa Fluor 488 (Abcam, ab150105, 1:200) secondary antibodies for 1 hour at room temperature. Sections were then rinsed three times for 10 minutes in PBS and then mounted with coverslips using VectaShield with DAPI (Vector Laboratories H‐1200).

### 2.3 Image capture and quantification

Histology images were observed on an optical microscope (Axio Imager; Zeiss), and fluorescent images were acquired using an Axio Observer D1 fluorescence microscope. Pictures were analyzed using image analysis software (ImageJ; National Institutes of Health) with thresholding set to identify the areas staining positive for lipid with Oil‐Red‐O and for fibrosis with Masson's trichrome. All pictures were assessed by two reviewers blinded to group status. Fat and fibrosis indices were calculated as the average percent of the area of tissue staining positive for fat or fibrosis of three representative ×20 image fields of view each from a different, randomly selected muscle section per donor. Representative image fields were chosen to capture the general composition and distribution of collagen staining fibers and fat droplets after Masson's trichrome and Oil‐Red‐O staining, respectively.

### 2.4 Cell isolation

Muscle specimens underwent digestion separately at 37°C in 0.2% collagenase for 90 minutes followed by 0.4% dispase for 30 minutes. The resulting suspensions were filtered through a 40 μm cell strainer, then pelleted and resuspended in FACS buffer (5% Fetal Bovine Serum in PBS). FAP and SC quantity were reported as a percentage of nondebris live cells, as determined using LIVE/DEAD Fixable Aqua Dead Cell Stain Kit (ThermoFisher) and their respective surface markers. Cells were sorting and quantified using FACS BD Aria II. FAPs were defined as CD31‐/CD45‐/CD29‐/CD56‐/PDGFRα+/CD184‐, and SCs were defined as CD31‐/CD45‐/CD29+/CD56+/PDGFRα‐/CD184+ as previously described.^29^, ^30^ Gates were set based on each antibodies' fluorescence minus one for every run. The antibodies used for FACS are as follows at concentration of 1 μg/mL per 1 × 10^6^ cells/mL: CD56 (Biolegend, Clone HCD56, #318304, FITC), CD29 (BD bioscience, Clone MAR4, #743783, Pacific Blue), CD184 (Biolegend, Clone 12G5, #306510, APC), PDGFRα (Biolegend, Clone 16A1, #323508, APC/CY7), CD31 (Biolegend, Clone WM59, #303122, BV605), and CD45 (Biolegend, Clone 2D1, #368524, BV605).

### 2.5 FAP and SC gene expression reverse transcription‐polymerase chain reaction

FAPs (CD31‐/CD45‐/CD29‐/CD56‐/PDGFRα+/CD184‐) and SCs (CD31‐/CD45‐CD29+/CD56+/PDGFRα‐/CD184+) from each of the 10 donor tissue were FACS sorted separately and directly into Trizol reagent (ThermoFisher) for total RNA extraction. The Transcriptor First Strand cDNA Synthesis Kit (Roche Applied Bioscience Inc.) was applied to synthesize cDNA. We performed reverse transcription‐polymerase chain reaction (RT‐PCR) using Fast SYBR Green Master Mix (Applied Biosystems) on a Viia7 Real Time Detection System (Applied Biosystems) to quantify the expression of genes. A total of 5n g of cDNA was used per reaction. Primer sequences of the genes tested are summarized in Supplemental Table S1. The expression level of each gene was normalized to that of the housekeeping gene of S26. Fold difference relative to hamstring controls was calculated using double delta cycle threshold.

### 2.6 Statistical analysis

*t*‐Tests were performed for statistical analysis to evaluate the differences in FAP and SC quantity and gene expression between FAPs and SCs taken from the multifidus and those taken from hamstring tissue. Prism 7 software (version 7.0a, GraphPad Software, Inc.) was used for statistical testing and figure creation. Data are presented as mean ± SD. Statistical corrections were employed using the Benjamini‐Hochberg method. Significance was defined as *P* < .05.

## 3 RESULTS

### 3.1 Demographics

The mean age of patients from which multifidus muscle was collected was 62.2 ± 17.6 years. Patient age range was 37 to 83 years. There were eight males and two female patients. The anterior cruciate ligament (ACL) reconstruction with hamstring autograft cohort consisted of three females and one male. The mean patient age was 60.5 ± 11.6 with a range of 47 to 74 years.

### 3.2 Multifidus muscle tissue contains higher fat content and fibrotic tissue

**FIGURE 1:**
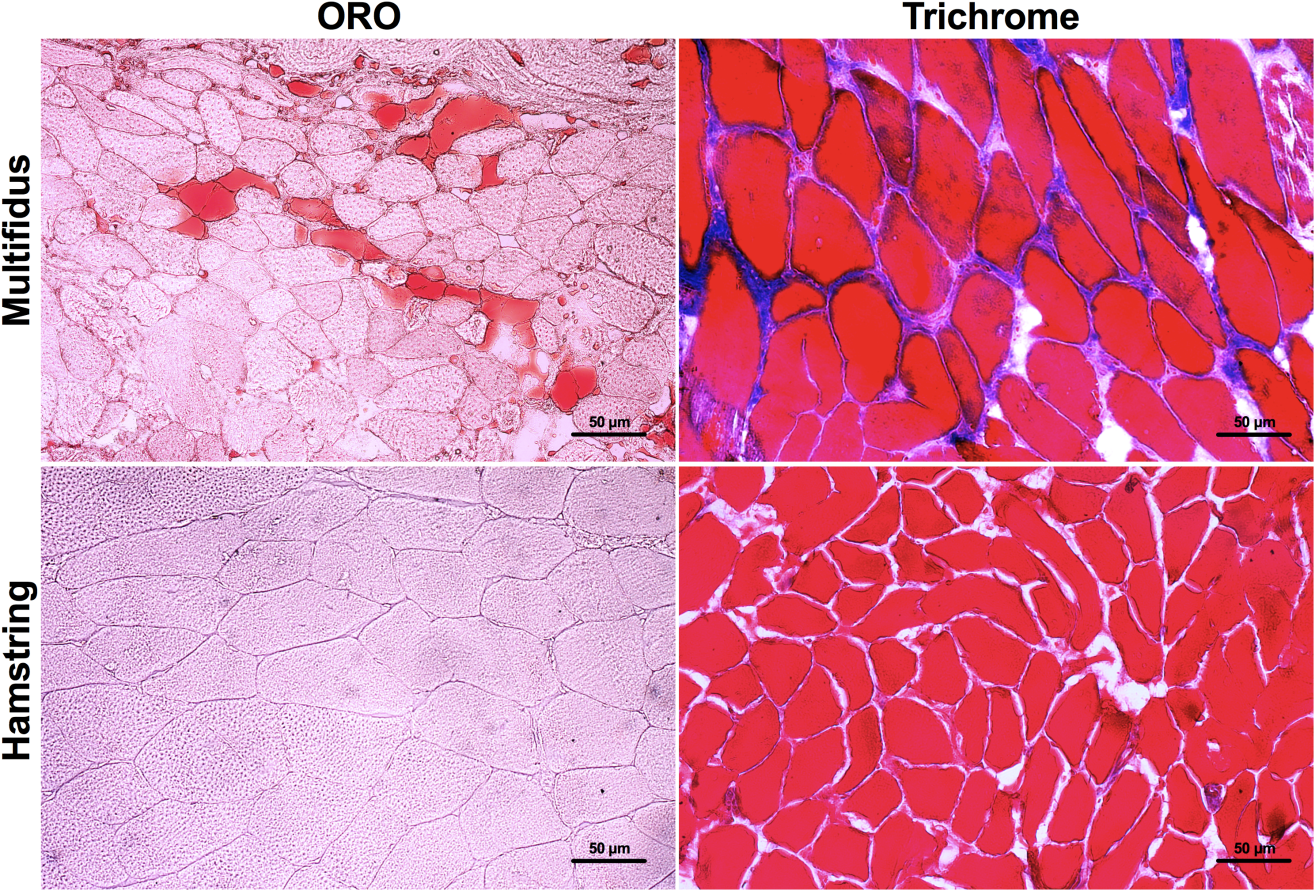
Representative histology images of multifidus and hamstring muscle specimens, ×20 magnification. Left, Oil‐Red‐O(ORO) staining for fatty infiltration (red). Right, Masson's trichrome staining for fibrosis (blue/purple)

Oil‐Red‐O and Masson's trichrome staining demonstrated extensive FI and fibrosis in multifidus muscle from patients with disc herniation (Figure 1). The quantified area composition of fat and fibrotic tissue accounted for 14.2% ± 7.4% and 14.8% ± 4.2%, respectively. The hamstring muscle did not contain appreciable levels of fat or fibrosis with values well below 1%.

### 3.3 Multifidus muscle contains a higher number of FAPs and SCs

**FIGURE 2:**
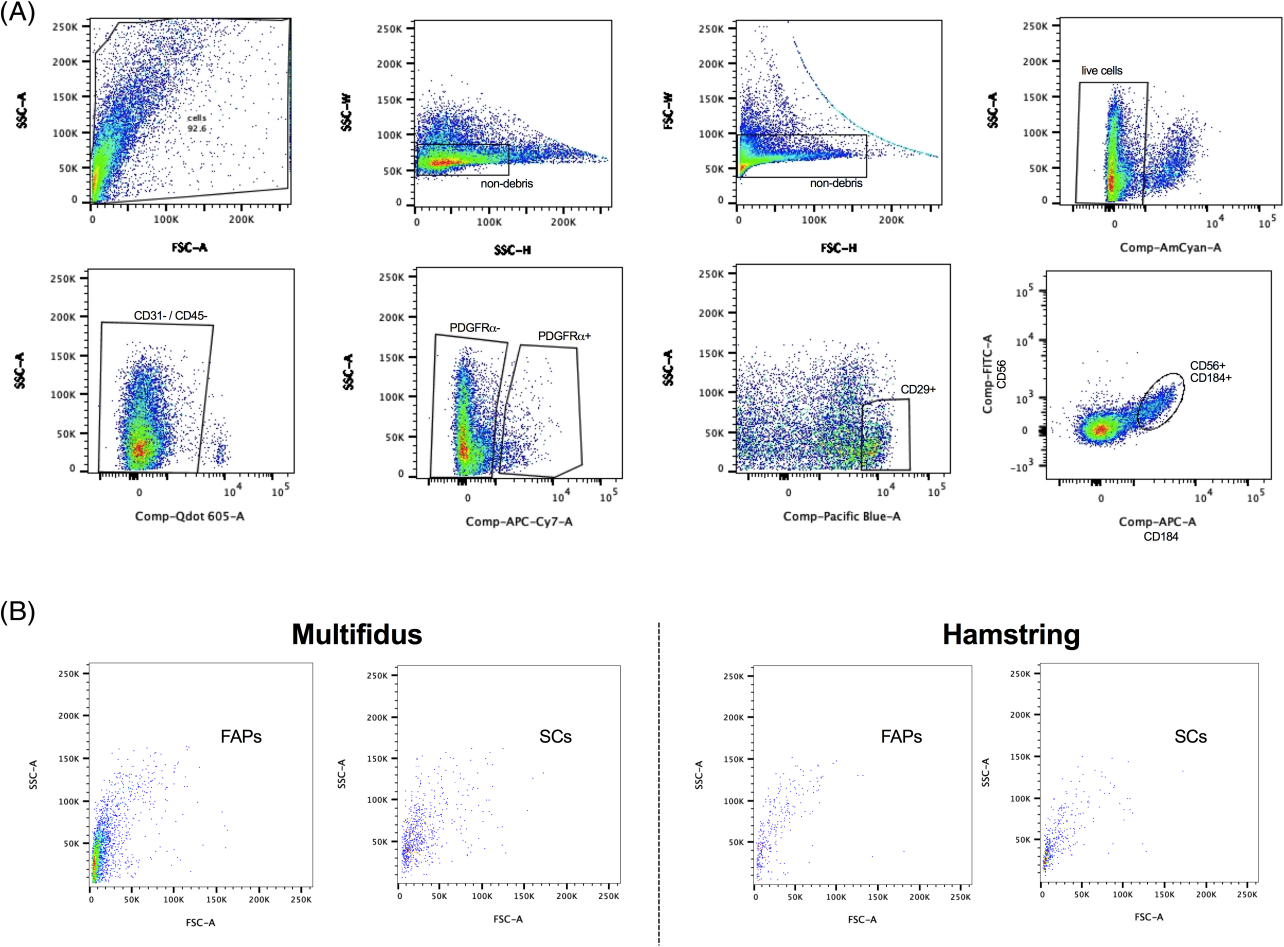
A, Full gating of FACS cell sorting. Gates were set based on each antibodies' FMO for every run. FAP and SC quantity were reported as a percentage of nondebris live cells. Sorting order is from top left to top right to bottom left to bottom right with the final FAP population defined as CD31‐/CD45‐/CD29‐/CD56‐/PDGFRα+/CD184‐, and final SC population defined as CD31‐/CD45‐/CD29+/CD56+/PDGFRα‐/CD184+. B, Forward and side scatter plots of final FAP and SC populations from the multifidus and hamstring. FAP, fibro‐adipogenic progenitor; FMO, fluorescence minus one; PDGFRα, platelet‐derived growth factor receptor alpha; SC, satellite cell

**FIGURE 3:**
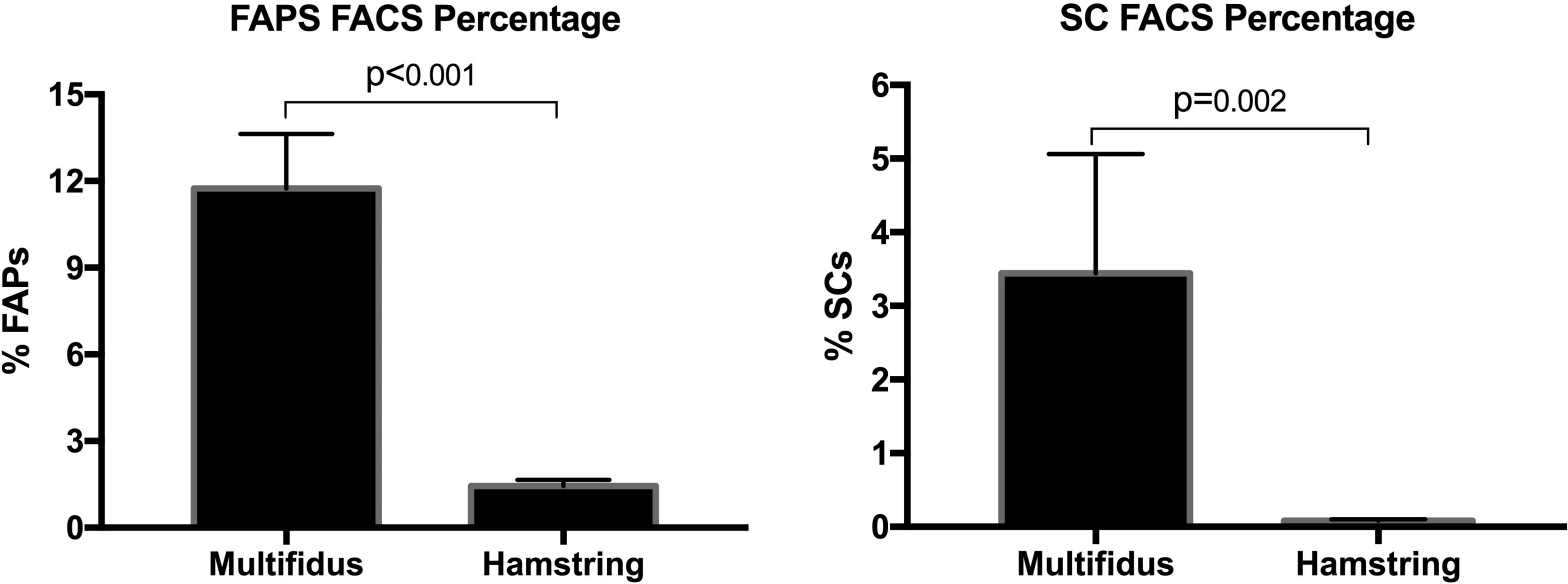
Percentages of FAPs (left) and SCs (right) within the multifidus and hamstring muscle as quantified using FACS cell sorting. FAP and SC quantity were reported as a percentage of non‐debris live cells. FAP, fibro‐adipogenic progenitor; SC, satellite cell

**FIGURE 4:**
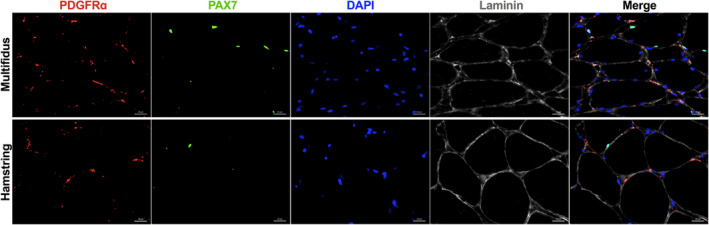
Representative immunostaining of multifidus and hamstring muscle demonstrating respective relative proportions of FAPs and SCs residing in the muscle, ×40 magnification, scale bar represents 20 μm. Red, PDGFRα ‐ FAP marker. Green, PAX7 ‐ SC marker. Blue, DAPI. Gray, laminin. FAP, fibro‐adipogenic progenitor; PDGFRα, platelet‐derived growth factor receptor alpha; SC, satellite cell

FACS sorting for FAPs and SCs from the muscle specimens were performed (Figure 2). There were significantly more FAPs in the multifidus than in the hamstring (11.7% ± 1.9% vs 1.4% ± 0.2%; *P* < .001) (Figure 3). Similarly, the multifidus contained significantly more SCs than the hamstring muscle (3.4% ± 1.6% vs 0.08% ± 0.02%; *P* = .002). In addition, immunostaining using antibodies against PDGFRα and Pax7 revealed large quantities of FAPs and SCs within the multifidus (Figure 4).

### 3.4 FAPs from multifidus express high levels of αSMA and adipogenic markers

**FIGURE 5:**
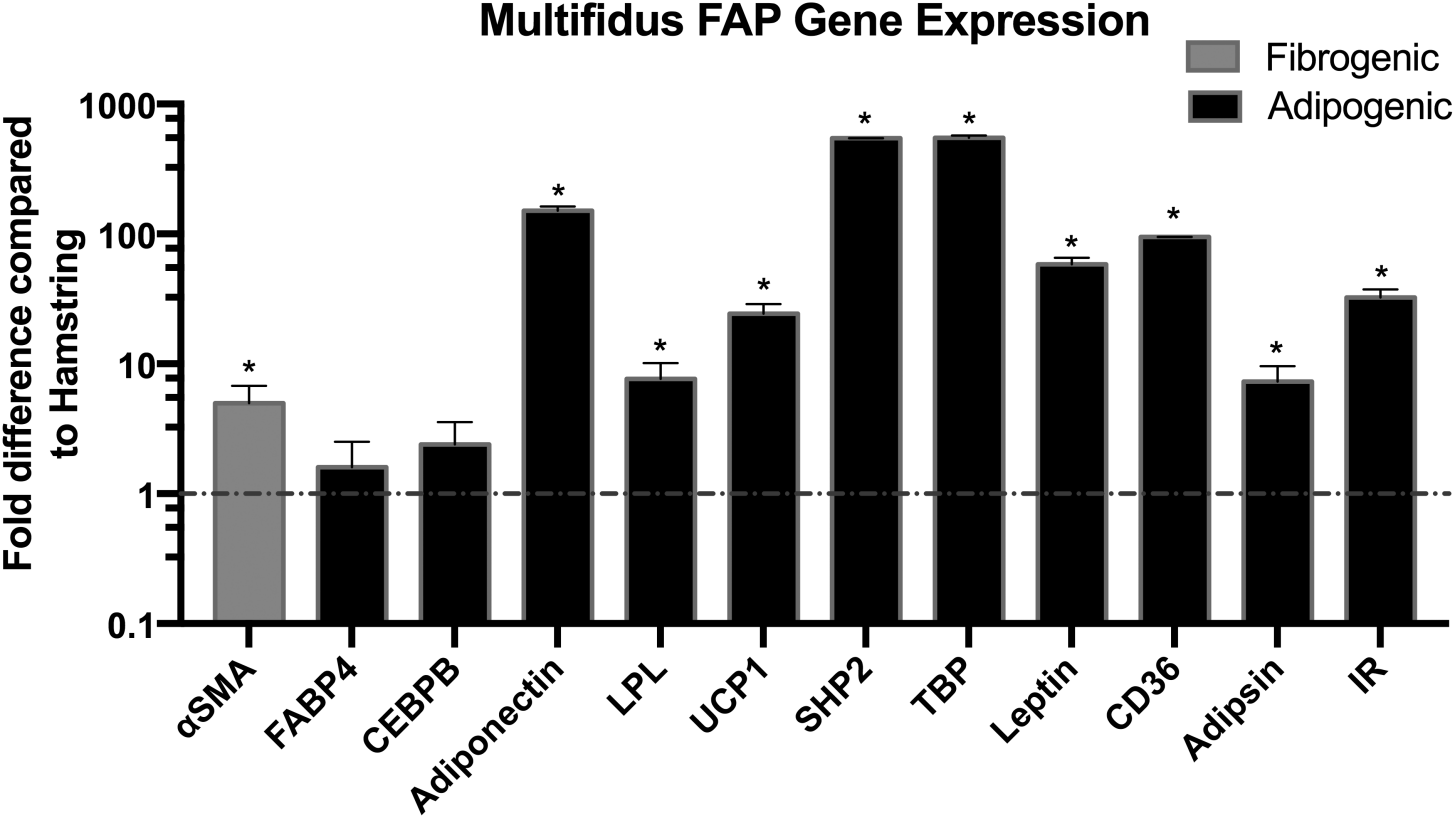
Comparative gene expression profile of FAPs from multifidus normalized to FAPs from hamstring. Gray bar designates fibrogenic gene expression. Black bars designate adipogenic gene expression. Y‐axis scale represents logarithmic fold difference. * denotes *P* < .05. FAP, fibro‐adipogenic progenitor

A panel of fibrogenic and adipogenic genes were analyzed using RT‐PCR for both FAPs harvested from the multifidus and those from the hamstring (Figure 5). FAPs from the multifidus demonstrated a significantly increased fold change of 5.0 ± 1.8 (*P* < .05) in αSMA expression, a fibrosis marker, when compared to those in the hamstring. Multiple adipogenic genes had significantly greater expression in the FAPs from the multifidus than the hamstring. In particular, adiponectin, Src homology domain‐containing protein tyrosine phosphatase 2, and TBP showed fold change increases of 150.9 ± 11.4, 545.5 ± 1.5, and 549.4 ± 23.3 (*P* < .05) in that order. The brown/beige fat marker uncoupling protein 1 (UCP1) was also significantly increased with a fold change of 24.3 ± 4.6 (*P* < .05).

### 3.5 SCs from multifidus display greater stem, proliferation, and differentiation gene expression

**FIGURE 6:**
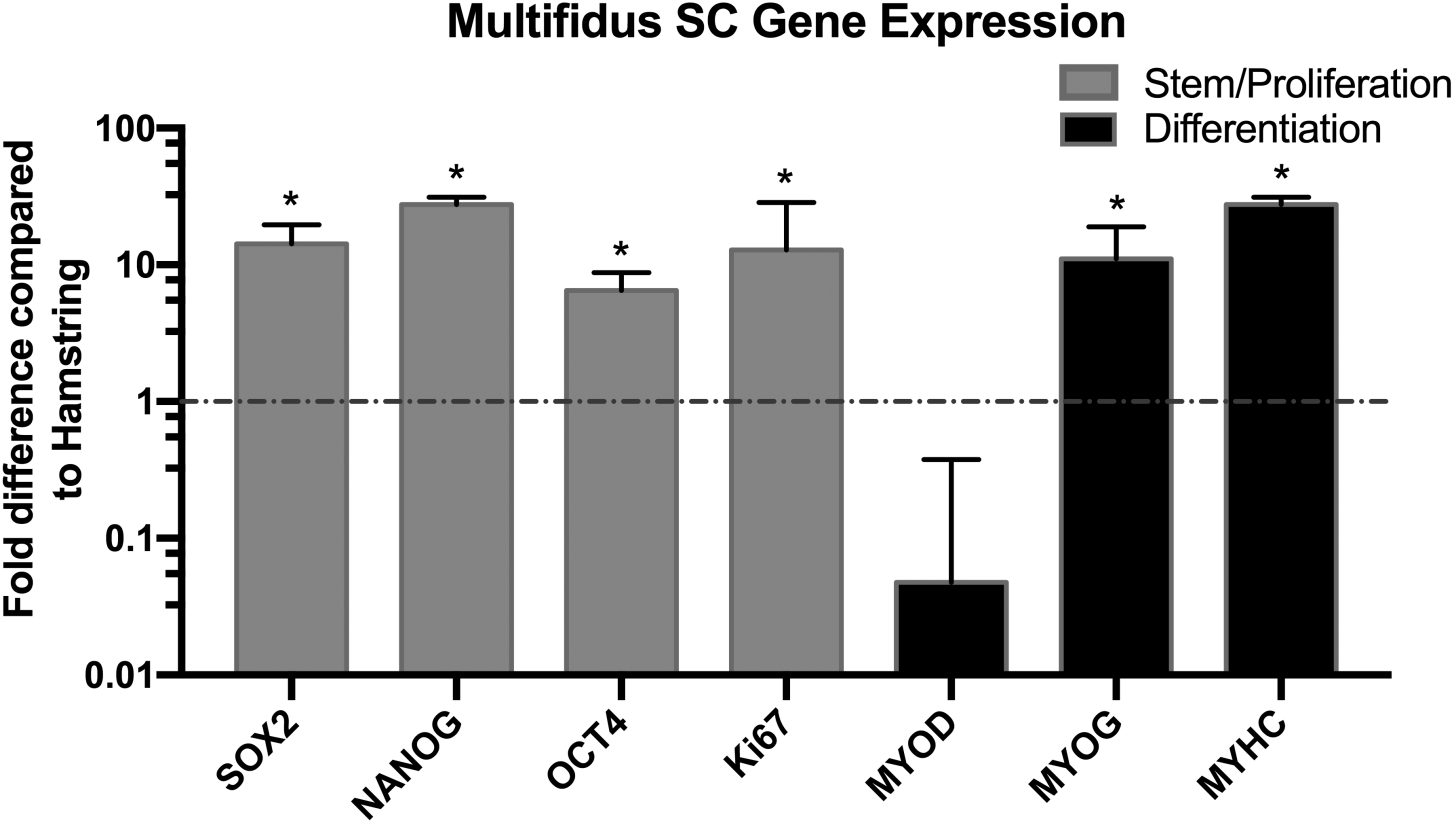
Comparative gene expression profile of SCs from multifidus normalized to SCs from hamstring. Gray bars designate stem/proliferation gene expression. Black bars designate differentiation gene expression. Y‐axis scale represents logarithmic fold difference. * denotes *P* < .05. SC, satellite cell

A panel of SC stem, proliferation, and differentiation genes were analyzed using RT‐PCR(Figure 6). Compared to those from the hamstring, SCs from the multifidus showed significantly higher expression of genes involved in preserving stem cell phenotypes. SOX2, NANOG, and OCT4 had fold change increases of 14.2 ± 5.3, 27.5 ± 3.5, and 6.4 ± 2.3 (*P* < .05) in that order. A significant fold increase in expression of Ki67, a marker of cell proliferation, was observed in SC from the multifidus. In addition, the myogenic differentiation genes *MYOG* and *MYHC* had significant fold increases of 11.0 ± 7.8 and 27.4 ± 3.5 (*P* < .05), respectively. Interestingly, *MYOD*, a major gene associated with initiation of SC myogenic differentiation, demonstrated a fold change reduction of 0.05 ± 0.3 in multifidus SCs compared to the hamstring, although this difference did not reach significance.

## 4 DISCUSSION

Our study demonstrates that multifidus tissue in lumbar disc herniation patients contains high levels of FI and fibrosis. We also demonstrated a markedly higher percentage of FAPs (11.7%) residing in the multifidus compared to hamstring control (1.4%) as well as elevated expression of αSMA, a fibrosis marker, and adipogenic genes. Studies examining the role of FAPs in FI and fibrosis of other muscle groups, such as the rotator cuff, have described similar findings.^31^, ^32^ Additionally, multifidus SCs were of greater number and displayed increased gene expression of stem, proliferation, and differentiation markers. Many studies have reported on the associations among painful disc herniation, the resulting denervation and disuse muscle injury, and the development of FI, fibrosis, and atrophy^20^, ^33^, ^34^, ^35^, ^36^ However, there is extremely limited literature on stem cell populations in paraspinal muscle and their relation to these degenerative features. To our knowledge, this study is the first to specifically examine both FAPs and SCs in the multifidus, and compare gene expression between the cell populations harvested from different muscle groups.

Our data regarding multifidus fat and fibrosis percentages is similar to that of other studies with histologic methods. One study that used histologic assessment of fat and fibrosis reported 11.7% adipose tissue and 26.1% fibrotic tissue in multifidus biopsies from patients with degenerative disc disease.^8^ Another recent study which evaluated fat and fibrosis percentages in multifidus biopsies for a cohort of surgical spine cases, including patients undergoing lumbar disc herniation decompressive surgery, reported a multifidus fat percentage of 14.3% ± 12.3% and a fibrosis percentage of 21.2% ± 12.7%.^37^ Most studies, however, quantify disc herniation paraspinal FI with magnetic resonance imaging (MRI) often using semiquantitative methods of which tend to have a wider range of values and greater averages.^4^, ^17^, ^38^ These differences in disease and quantification methods make direct comparisons challenging as MRI FI assessment often includes fat consolidations surrounding muscle fascicles and compartments not normally captured in small multifidus histology sections. Histologic evaluation employed in our study likely provides a more accurate assessment of intrafiber and interfiber FI of the multifidus than macroscopic imaging techniques.

The mechanism underlying the high quantity and elevated αSMA and adipogenic gene expression of FAPs in the multifidus, as found in our study, may be multifactorial. Baseline quantitative and qualitative differences in FAPs based on anatomic location are one possibility. Previous work by our lab demonstrated that uninjured mouse paraspinal muscles had higher concentrations of FAPs that exhibited increased proliferation and adipogenic potency compared to FAPs taken from hind limb muscles.^39^ Interestingly, greater adipogenic gene expression was also noted in the human FAPs from the multifidus in this current study. Other studies have also detailed baseline differences in FAP populations.^40^, ^41^, ^42^, ^43^, ^44^ Davies et al observed upregulated adipogenic genes and increased FI in the rotator cuff of mice compared to the gastrocnemius after analogous tendon‐nerve transection injury at each site.^45^ These results taken to together may provide some explanation for paraspinal muscle's proclivity to develop FI. FAPs residing is specific regions may be more primed for adipogenic differentiation than those in other muscle groups.

Beyond possible inherent differences between FAP populations within the multifidus and hamstring, the disuse‐denervation injury specific features of disc herniation may also play a role. Pagano et al demonstrated upregulated expression of PDGFRα and adipogenic markers as well as increased intramuscular FI in a short‐term disuse model.^46^ In an amyotrophic lateral sclerosis mouse model that resulted in diffuse denervation, increases in muscle atrophy, fibrosis, and FAP number were observed.^47^ In addition, Madaro et al demonstrated progressive fibrosis and FAP accumulation within hind limb muscles after sciatic nerve transection compared to an intramuscular cardiotoxin injection injury.^48^ These denervated FAPs (FAPDENs) displayed increased proliferation and upregulation of genes associated with muscle atrophy, such as *IL‐6* and *STAT3*.^49^, ^50^, ^51^, ^52^ FAPDENs cocultured with myoblasts reduced myoblast fusion and fiber diameter. Furthermore, transplantation of FAPDENs into healthy muscle in vivo, decreased cross‐sectional area of the surrounding muscle fibers. These findings suggest that elevated concentrations of FAPs in the multifidus of disc herniation patients may be a result of the disuse‐denervation sequelae that expands the pool of fibroblastic and adipogenic‐cued cells while potentially promoting downstream muscle atrophy via denervation specific FAP reprogramming.

Our study demonstrated markedly elevated percentages of SCs within the multifidus compared to the hamstring. Shahidi et al examined SCs in human multifidus muscle biopsies from patients with degenerative lumbar disc disease and found SCs at a rate of 13 ± 9 per 100 muscle fibers.^8^ Due to differences in disease type and quantification methods, a direct comparison to our study is not possible. However, discovering a large quantity of SCs in our study residing in muscle plagued by atrophy is seemingly paradoxical and not fully understood at this time. It is possible that the large amounts of SCs present in the multifidus are simply reflective of higher baseline concentrations as has been reported in literature regarding other muscle groups.^53^, ^54^ Alternatively, active degeneration of the muscle may trigger SC expansion; however, their inability to reliably regenerate the multifidus may point to overwhelming perturbations in the SC niche that hinders their effectiveness.

The differences in gene expression of stem, proliferation, and differentiation markers between SCs from multifidus and the hamstring detailed in our study further raise the possibility that changes in overall SC activation could play a role in the mismatch of increased SC quantity and atrophy. Upon injury, SCs proliferate and differentiate asymmetrically of which the majority of daughter cells continue toward myoblasts that form new myofibers. A small fraction of these daughter cells replenish the SC pool and retain their stemness as designated by continued Pax7 positivity. Studies have found that after denervation injury, SC populations within muscle increase, although this increase may be somewhat dependent on the duration of denervation.^55^, ^56^, ^57^, ^58^ Our study reveals upregulation of genes that preserve stemness and promote proliferation.^59^, ^60^ Interestingly, we observed a trend toward decreased expression of MYOD, a critical gene involved in the initial steps of SC activation and differentiation.^61^, ^62^ Although we see increases in other downstream markers of differentiation, it may be that concurrent reduction in MYOD expression tilts the balance of SC activation toward proliferation. Additionally, Borisov et al showed impaired terminal differentiation of myoblast in denervated muscle and that nascent myotubes were often confined within a fibrotic matrix, which could further contribute to the imbalance.^63^, ^64^ This potentially impaired transition of denervation‐activated SCs to terminally differentiated myotubes may result in simultaneous SC accumulation and atrophy progression. Regardless of the mechanism of SC accumulation, the findings outlined in our study may provide new strategies to drive multifidus muscle regeneration by utilizing this large pool of myogenic progenitors.

We also happened to observe elevated levels of UCP1 expression in FAPs from the multifidus compared to those from the hamstring. FAPs have been shown to adopt a brown/beige fat (BAT) phenotype characterized by elevated expression of UCP1 that represents a more metabolically active tissue than its white fat (WAT) counterpart that comprises the majority of muscle FI.^24^, ^65^, ^66^ BAT has been shown to have positive effects on myogenesis likely through paracrine signaling and “batokine” factor secretion.^67^, ^68^, ^69^, ^70^ Transplantation of BAT in rotator cuff muscles after cardiotoxin injury increased muscle mass, contractile force, and fiber cross‐sectional area.^71^ Wang et al demonstrated that reversal of rotator cuff FI in mice after suprascapular nerve compression and release likely involved the “browning” of white adipocytes.^66^ Our findings of high multifidus FAP percentage and UCP1 expression may represent an opportunity for novel therapeutic approaches to reduce the amount of FI and curtail muscle atrophy by driving FAPs into the more beneficial BAT phenotype. Future studies are warranted to evaluate pharmacologic and transplantation strategies to increase adoption of the BAT phenotype by FAPs in paraspinal muscle and assess effects on FI, muscle quality, and function.

There are limitations to our study. We compare multifidus muscle after disc herniation to hamstring muscle. It is possible that ACL injury results local inflammation, disuse injury, and biomechanical changes that could impact hamstring morphology and stem cell populations, as has been demonstrated within the vastus lateralis by Fry et al^72^ Ideally, uninjured multifidus would be used as a control for such a study. However, due to the nature of human clinical research, procuring uninjured multifidus muscle from patients not undergoing a concurrent spine surgery was not feasible at this time. Future studies will attempt to obtain samples from other sources to allow for more robust direct comparisons. Additionally, procuring samples from patients undergoing surgery for different spinal conditions could further elucidate disc herniation specific features of FAPs and SCs related to paraspinal muscle degeneration Studies have shown that αSMA expression and collagen gene expression is significantly regulated by the TGFβ‐SMAD signaling pathway.^31^, ^73^, ^74^ Other studies have evaluated FAP αSMA expression in the setting of fibrogenesis and have detailed αSMA expression in myofibroblasts, which bear close similarity to FAPs.^74^, ^75^ However, there are likely additional genes involved in multifidus FAP‐derived fibrosis other than αSMA examined in our study. Future studies will aim to expand the number of genes tested to further characterize FAP‐fibrosis pathways in paraspinal muscle. Finally, the age range of the patients in our study is relatively wide. Age may impact baseline levels of stem cell populations as well as their responses to a variety of injury types, although we observed relatively narrow SC and FAP percentage standard deviations in this study. Future work will aim to expand the sample size and overall scope of the present study to better characterize the relationships between age, gene expression, muscle stem cell activity, and muscle pathology in disc herniation patients. These types of studies can identify potential therapeutic targets and help distinguish for which patient subsets they would be most effective.

In conclusion, this study demonstrates that multifidus muscle in patients undergoing surgery for disc herniation contain large quantities of FAPs and SCs which display different gene expression profiles compared to their counterparts within hamstring tissue. These results may help explain the multifidus' propensity to form FI and fibrosis and can shed light on novel strategies to target this cell population to improve muscle quality. In addition, the discovery of a large SC population within the multifidus in these patients who often suffer from muscle atrophy may prove useful in developing cell‐based approaches that harness the SC pool to mitigate or reverse degeneration.

## CONFLICT OF INTEREST

O. A., A. M. I., M. L., H. Z., H. T. K, X. L., B. T. F., and J. B. F., their immediate family, and any research foundation with which they are affiliated did not receive any financial payments or other benefits from any commercial entity related to the subject of this article. A. A. T. is a consultant for Alphatec and DePuy Spine, and participates in the development of educational content for the Journal of Bone and Joint Surgery, all of which are unrelated to the submitted work. A. C. is a consultant for NuVasive, unrelated to the submitted work.

## AUTHOR CONTRIBUTIONS

Obiajulu Agha: experimental design, data acquisition and analysis, manuscript writing, review, and revision. Andreas Mueller‐Immergluck: experimental design, data acquisition and analysis, and manuscript review and revision. Mengyao Liu: experimental design, data acquisition and analysis, and manuscript review and revision. He Zhang: experimental design, data acquisition and analysis, and manuscript review and revision. Alekos A. Theologis: experimental design, muscle tissue harvest, and manuscript review and revision. Aaron Clark: experimental design, muscle tissue harvest, and manuscript review and revision. Hubert T. Kim: coprincipal investigator, experimental design, and manuscript review and revision. Xuhui Liu: coprincipal investigator, experimental design, and manuscript review and revision. Brian T. Feeley: coprincipal investigator, experimental design, and manuscript review and revision. Jeannie F. Bailey: experimental design, data acquisition and analysis, and manuscript review and revision. All authors have read and approved the submitted manuscript.

## Supporting information


**Table S1** Supporting information

## References

[1] Ringwalt C , Gugelmann H , Garrettson M , et al. Differential prescribing of opioid analgesics according to physician specialty for Medicaid patients with chronic noncancer pain diagnoses. Pain Res Manag. 2014;19:179‐185. 10.1155/2014/857952. 24809067 PMC4158932

[2] Macintosh JE , Bogduk N . The biomechanics of the lumbar multifidus. Clin Biomech. 1986;1:205‐213. 10.1016/0268-0033(86)90147-6. 23915551

[3] Panjabi M , Abumi K , Duranceau J , et al. Spinal stability and intersegmental muscle forces. A biomechanical model. Spine. 1989;14:194‐200. 10.1097/00007632-198902000-00008. 2922640

[4] Cooley JR , Walker BF , E MA , et al. Relationships between paraspinal muscle morphology and neurocompressive conditions of the lumbar spine: a systematic review with meta‐analysis. BMC Musculoskelet Disord. 2018;19:351 10.1186/s12891-018-2266-5. 30261870 PMC6161433

[5] Fortin M , Lazary A , Varga PP , et al. Association between paraspinal muscle morphology, clinical symptoms and functional status in patients with lumbar spinal stenosis. Eur Spine J. 2017;26:2543‐2551. 10.1007/s00586-017-5228-y. 28748488

[6] Faur C , Patrascu JM , Haragus H , Anglitoiu B . Correlation between multifidus fatty atrophy and lumbar disc degeneration in low back pain. BMC Musculoskelet Disord. 2019;20:414 10.1186/s12891-019-2786-7. 31488112 PMC6729014

[7] Wajchenberg M , Martins DE , Luciano Rde P , et al. Histochemical analysis of paraspinal rotator muscles from patients with adolescent idiopathic scoliosis: a cross‐sectional study. Medicine. 2015;94:e598 10.1097/md.0000000000000598. 25715269 PMC4554143

[8] Shahidi B , Hubbard JC , Gibbons MC , et al. Lumbar multifidus muscle degenerates in individuals with chronic degenerative lumbar spine pathology. J Orthop Res. 2017;35:2700‐2706. 10.1002/jor.23597. 28480978 PMC5677570

[9] Goubert D , de Pauw R , Meeus M , et al. Lumbar muscle structure and function in chronic versus recurrent low back pain: a cross‐sectional study. Spine J. 2017;17:1285‐1296. 10.1016/j.spinee.2017.04.025. 28456669

[10] Goubert D , Oosterwijck JV , Meeus M , Danneels L . Structural changes of lumbar muscles in non‐specific low back pain: a systematic review. Pain Physician. 2016;19:E985‐e1000. 27676689

[11] Teichtahl AJ , Urquhart DM , Wang Y , et al. Fat infiltration of paraspinal muscles is associated with low back pain, disability, and structural abnormalities in community‐based adults. Spine J. 2015;15:1593‐1601. 10.1016/j.spinee.2015.03.039. 25828477

[12] Bailey JF , Miller SL , Khieu K , et al. From the international space station to the clinic: how prolonged unloading may disrupt lumbar spine stability. Spine J. 2018;18:7‐14. 10.1016/j.spinee.2017.08.261. 28962911 PMC6339989

[13] Ranger TA , Cicuttini FM , Jensen TS , et al. Are the size and composition of the paraspinal muscles associated with low back pain? A systematic review. Spine J. 2017;17:1729‐1748. 10.1016/j.spinee.2017.07.002. 28756299

[14] Kalichman L , Carmeli E , Been E . The association between imaging parameters of the paraspinal muscles, spinal degeneration, and low back pain. Biomed Res Int. 2017;2017:2562957 10.1155/2017/2562957. 28409152 PMC5376928

[15] Battie MC , Niemelainen R , Gibbons LE , et al. Is level‐ and side‐specific multifidus asymmetry a marker for lumbar disc pathology? Spine J. 2012;12:932‐939. 10.1016/j.spinee.2012.08.020. 23084154

[16] Kulig K , Scheid AR , Beauregard R , Popovich JM Jr , Beneck GJ , Colletti PM . Multifidus morphology in persons scheduled for single‐level lumbar microdiscectomy: qualitative and quantitative assessment with anatomical correlates. Am J Phys Med Rehabil. 2009;88:355‐361. 19630124 10.1097/phm.0b013e31819c506d

[17] Fortin M , Lazary A , Varga PP , et al. Paraspinal muscle asymmetry and fat infiltration in patients with symptomatic disc herniation. Eur Spine J. 2016;25:1452‐1459. 10.1007/s00586-016-4503-7. 26957101

[18] Hodges P , Holm AK , Hansson T , Holm S . Rapid atrophy of the lumbar multifidus follows experimental disc or nerve root injury. Spine. 2006;31:2926‐2933. 10.1097/01.brs.0000248453.51165.0b. 17139223

[19] Lehto M , Hurme M , Alaranta H , et al. Connective tissue changes of the multifidus muscle in patients with lumbar disc herniation. An immunohistologic study of collagen types i and iii and fibronectin. Spine. 1989;14:302‐309. 10.1097/00007632-198903000-00010. 2711245

[20] Sun D , Liu P , Cheng J , Ma Z , Liu J , Qin T . Correlation between intervertebral disc degeneration, paraspinal muscle atrophy, and lumbar facet joints degeneration in patients with lumbar disc herniation. BMC Musculoskelet Disord. 2017;18:167‐167. 10.1186/s12891-017-1522-4. 28427393 PMC5399427

[21] Wesselink E , de Raaij E , Pevenage P , et al. Fear‐avoidance beliefs are associated with a high fat content in the erector spinae: a 1.5 tesla magnetic resonance imaging study. Chiropr Man Therap. 2019;27:14 10.1186/s12998-019-0234-2. PMC6419476 30918625

[22] Willemink MJ , van Es HW , Helmhout PH , Diederik AL , Kelder JC , van Heesewijk JPM . The effects of dynamic isolated lumbar extensor training on lumbar multifidus functional cross‐sectional area and functional status of patients with chronic nonspecific low back pain. Spine. 2012;37:E1651‐E1658. 10.1097/BRS.0b013e318274fb2f. 23023592

[23] Shahtahmassebi B , Hebert JJ , Stomski NJ , Hecimovich M , Fairchild TJ . The effect of exercise training on lower trunk muscle morphology. Sports Med. 2014;44:1439‐1458. 10.1007/s40279-014-0213-7. 25015476

[24] Joe AW , Yi L , Natarajan A , et al. Muscle injury activates resident fibro/adipogenic progenitors that facilitate myogenesis. Nat Cell Biol. 2010;12:153‐163. 10.1038/ncb2015. 20081841 PMC4580288

[25] Quarta M , Cromie M , Chacon R , et al. Bioengineered constructs combined with exercise enhance stem cell‐mediated treatment of volumetric muscle loss. Nat Commun. 2017;8:15613 10.1038/ncomms15613. 28631758 PMC5481841

[26] Biferali B , Proietti D , Mozzetta C , Madaro L . Fibro–adipogenic progenitors cross‐talk in skeletal muscle: the social network. Front Physiol. 2019;10 1074 10.3389/fphys.2019.01074. 31496956 PMC6713247

[27] Uezumi A , Fukada S , Yamamoto N , Takeda S' , Tsuchida K . Mesenchymal progenitors distinct from satellite cells contribute to ectopic fat cell formation in skeletal muscle. Nat Cell Biol. 2010;12:143‐152. 10.1038/ncb2014. 20081842

[28] Uezumi A , Ito T , Morikawa D , et al. Fibrosis and adipogenesis originate from a common mesenchymal progenitor in skeletal muscle. J Cell Sci. 2011;124:3654‐3664. 10.1242/jcs.086629. 22045730

[29] Uezumi A , Nakatani M , Ikemoto‐Uezumi M , et al. Cell‐surface protein profiling identifies distinctive markers of progenitor cells in human skeletal muscle. Stem Cell Rep. 2016;7:263‐278. 10.1016/j.stemcr.2016.07.004. PMC4983081 27509136

[30] Uezumi A , Ikemoto‐Uezumi M , Tsuchida K . Roles of nonmyogenic mesenchymal progenitors in pathogenesis and regeneration of skeletal muscle. Front Physiol. 2014;5:68 10.3389/fphys.2014.00068. 24605102 PMC3932482

[31] Davies MR , Liu X , Lee L , et al. TGF‐beta small molecule inhibitor SB431542 reduces rotator cuff muscle fibrosis and fatty infiltration by promoting fibro/Adipogenic progenitor apoptosis. PLoS One. 2016;11:e0155486 10.1371/journal.pone.0155486. 27186977 PMC4871364

[32] Liu X , Ning AY , Chang NC , et al. Investigating the cellular origin of rotator cuff muscle fatty infiltration and fibrosis after injury. Muscles Ligaments Tendons J. 2016;6:6‐15. 10.11138/mltj/2016.6.1.006. 27331027 PMC4915463

[33] Ross GB , Mavor M , Brown SH , et al. The effects of experimentally induced low back pain on spine rotational stiffness and local dynamic stability. Ann Biomed Eng. 2015;43:2120‐2130. 10.1007/s10439-015-1268-9. 25663629

[34] Ross GB , Sheahan PJ , Mahoney B , Gurd BJ , Hodges PW , Graham RB . Pain catastrophizing moderates changes in spinal control in response to noxiously induced low back pain. J Biomech. 2017;58:64‐70. 10.1016/j.jbiomech.2017.04.010. 28460690

[35] Burkhart K , Allaire B , Bouxsein ML . Negative effects of long‐duration spaceflight on paraspinal muscle morphology. Spine. 2019;44:879‐886. 10.1097/brs.0000000000002959. 30624302

[36] Ryall JG , Schertzer JD , Lynch GS . Cellular and molecular mechanisms underlying age‐related skeletal muscle wasting and weakness. Biogerontology. 2008;9:213‐228. 10.1007/s10522-008-9131-0. 18299960

[37] Shahidi B , Fisch KM , Gibbons MC , Ward SR . Increased fibrogenic gene expression in multifidus muscles of patients with chronic versus acute lumbar spine pathology. Spine. 2020;45:E189‐E195. 10.1097/brs.0000000000003243. 31513095 PMC6994378

[38] Kong B‐J , Lim J‐S , Kim K . A study on dispersion and rate of fat infiltration in the lumbar spine of patients with herniated nucleus polpusus. J Phys Ther Sci. 2014;26:37‐40. 10.1589/jpts.26.37. 24567672 PMC3927038

[39] Liu M , Lee C , Bertoy L , et al. Fiebro adipogenic progenitor (FAPs) populations differ across skeletal muscle. Paper presented at Orthopaedic Research Society Annual Meeting. Austin, TX; 2019.

[40] Lemos DR , Paylor B , Chang C , Sampaio A , Underhill TM , Rossi FMV . Functionally convergent white adipogenic progenitors of different lineages participate in a diffused system supporting tissue regeneration. Stem Cells. 2012;30:1152‐1162. 10.1002/stem.1082. 22415977

[41] Paylor B , Joe AW , Rossi FM , et al. In vivo characterization of neural crest‐derived fibro/adipogenic progenitor cells as a likely cellular substrate for craniofacial fibrofatty infiltrating disorders. Biochem Biophys Res Commun. 2014;451:148‐151. 10.1016/j.bbrc.2014.07.089. 25073114

[42] Stumm J , Vallecillo‐Garcia P , Vom Hofe‐Schneider S , et al. Odd skipped‐related 1 (Osr1) identifies muscle‐interstitialfibro‐adipogenic progenitors (FAPs) activated by acute injury. Stem Cell Res. 2018;32:8‐16. 10.1016/j.scr.2018.08.010. 30149291

[43] Murphy MM , Lawson JA , Mathew SJ , Hutcheson DA , Kardon G . Satellite cells, connective tissue fibroblasts and their interactions are crucial for muscle regeneration. Development. 2011;138:3625‐3637. 10.1242/dev.064162. 21828091 PMC3152921

[44] Wosczyna MN , Biswas AA , Cogswell CA , Goldhamer DJ . Multipotent progenitors resident in the skeletal muscle interstitium exhibit robust BMP‐dependent osteogenic activity and mediate heterotopic ossification. J Bone Miner Res. 2012;27:1004‐1017. 10.1002/jbmr.1562. 22307978 PMC3361573

[45] Davies MR , Ravishankar B , Laron D , Kim HT , Liu X , Feeley BT . Rat rotator cuff muscle responds differently from hindlimb muscle to a combined tendon‐nerve injury. J Orthop Res. 2015;33:1046‐1053. 10.1002/jor.22864. 25974842

[46] Pagano AF , Brioche T , Arc‐Chagnaud C , Demangel R , Chopard A , Py G . Short‐term disuse promotes fatty acid infiltration into skeletal muscle. J Cachexia Sarcopenia Muscle. 2018;9:335‐347. 10.1002/jcsm.12259. 29248005 PMC5879967

[47] Gonzalez D , Contreras O , Rebolledo DL , Espinoza JP , van Zundert B , Brandan E . ALS skeletal muscle shows enhanced TGF‐beta signaling, fibrosis and induction of fibro/adipogenic progenitor markers. PLoS One. 2017;12:e0177649 10.1371/journal.pone.0177649. 28520806 PMC5433732

[48] Madaro L , Passafaro M , Sala D , et al. Denervation‐activatedSTAT3‐IL‐6 signalling in fibro‐adipogenic progenitors promotes myofibres atrophy and fibrosis. Nat Cell Biol. 2018;20:917‐927. 10.1038/s41556-018-0151-y. 30050118 PMC6145844

[49] Bonetto A , Aydogdu T , Kunzevitzky N , et al. STAT3 activation in skeletal muscle links muscle wasting and the acute phase response in cancer cachexia. PLoS One. 2011;6:e22538 10.1371/journal.pone.0022538. 21799891 PMC3140523

[50] Sala D , Sacco A . Signal transducer and activator of transcription 3 signaling as a potential target to treat muscle wasting diseases. Curr Opin Clin Nutr Metab Care. 2016;19:171‐176. 10.1097/mco.0000000000000273. 27023048 PMC4866604

[51] Bonetto A , Aydogdu T , Jin X , et al. JAK/STAT3 pathway inhibition blocks skeletal muscle wasting downstream of IL‐6 and in experimental cancer cachexia. Am J Physiol Endocrinol Metab. 2012;303:E410‐E421. 10.1152/ajpendo.00039.2012. 22669242 PMC3423125

[52] Haddad F , Zaldivar F , Cooper DM , Adams GR . 2005. IL‐6‐induced skeletal muscle atrophy. J Appl Physiol. 1985;98:911‐917. 10.1152/japplphysiol.01026.2004. 15542570

[53] Schmalbruch H , Hellhammer U . The number of nuclei in adult rat muscles with special reference to satellite cells. Anat Rec. 1977;189:169‐175. 10.1002/ar.1091890204. 911042

[54] Mackey AL , Kjaer M , Charifi N , et al. Assessment of satellite cell number and activity status in human skeletal muscle biopsies. Muscle Nerve. 2009;40:455‐465. 10.1002/mus.21369. 19705426

[55] Hanzlikova V , Mackova EV , Hnik P . Satellite cells of the rat soleus muscle in the process of compensatory hypertrophy combined with denervation. Cell Tissue Res. 1975;160:411‐421. 10.1007/bf00222049. 1149125

[56] Snow MH . A quantitative ultrastructural analysis of satellite cells in denervated fast and slow muscles of the mouse. Anat Rec. 1983;207:593‐604. 10.1002/ar.1092070407. 6670756

[57] Viguie CA , Lu DX , Huang SK , Rengen H , Carlson BM . Quantitative study of the effects of long‐term denervation on the extensor digitorum longus muscle of the rat. Anat Rec. 1997;248:346‐354. 10.1002/(sici)1097-0185(199707)248:3<346::Aid-ar7>3.0.Co;2-n. 9214552

[58] Rodrigues Ade C , Schmalbruch H . Satellite cells and myonuclei in long‐term denervated rat muscles. Anat Rec. 1995;243:430‐437. 10.1002/ar.1092430405. 8597289

[59] Niwa H . How is pluripotency determined and maintained? Development. 2007;134:635‐646. 10.1242/dev.02787. 17215298

[60] Sun X , Kaufman PD . Ki‐67: more than a proliferation marker. Chromosoma. 2018;127:175‐186. 10.1007/s00412-018-0659-8. 29322240 PMC5945335

[61] Cornelison DD , Olwin BB , Rudnicki MA , et al. MyoD(−/−) satellite cells in single‐fiber culture are differentiation defective and MRF4 deficient. Dev Biol. 2000;224:122‐137. 10.1006/dbio.2000.9682. 10926754

[62] Yamamoto M , Legendre NP , Biswas AA , et al. Loss of MyoD and Myf5 in skeletal muscle stem cells results in altered myogenic programming and failed regeneration. Stem Cell Rep. 2018;10:956‐969. 10.1016/j.stemcr.2018.01.027. PMC5918368 29478898

[63] Borisov AB , Dedkov EI , Carlson BM . Differentiation of activated satellite cells in denervated muscle following single fusions in situ and in cell culture. Histochem Cell Biol. 2005;124:13‐23. 10.1007/s00418-005-0012-1. 16001203

[64] Borisov AB , Dedkov EI , Carlson BM . Abortive myogenesis in denervated skeletal muscle: differentiative properties of satellite cells, their migration, and block of terminal differentiation. Anat Embryol. 2005;209:269‐279. 10.1007/s00429-004-0429-7. 15761724

[65] Gorski T , Mathes S , Krutzfeldt J . Uncoupling protein 1 expression in adipocytes derived from skeletal muscle fibro/adipogenic progenitors is under genetic and hormonal control. J Cachexia Sarcopenia Muscle. 2018;9:384‐399. 10.1002/jcsm.12277. 29399988 PMC5879989

[66] Wang Z , Feeley BT , Kim HT , Liu X . Reversal of fatty infiltration after suprascapular nerve compression release is dependent on UCP1 expression in mice. Clin Orthop Relat Res. 2018;476:1665‐1679. 10.1097/corr.0000000000000335. 30020151 PMC6259770

[67] Chen Y , Ikeda K , Yoneshiro T , et al. Thermal stress induces glycolytic beige fat formation via a myogenic state. Nature. 2019;565:180‐185. 10.1038/s41586-018-0801-z. 30568302 PMC6328316

[68] Kong X , Yao T , Zhou P , et al. Brown adipose tissue controls skeletal muscle function via the secretion of myostatin. Cell Metab. 2018;28:631‐643.e633. 10.1016/j.cmet.2018.07.004. 30078553 PMC6170693

[69] Braga M , Reddy ST , Vergnes L , et al. Follistatin promotes adipocyte differentiation, browning, and energy metabolism. J Lipid Res. 2014;55:375‐384. 10.1194/jlr.M039719. 24443561 PMC3934723

[70] Singh R , Braga M , Pervin S . Regulation of brown adipocyte metabolism by myostatin/follistatin signaling. Front Cell Dev Biol. 2014;2:60 10.3389/fcell.2014.00060. 25364764 PMC4207030

[71] Bryniarski AR , Meyer GA . Brown fat promotes muscle growth during regeneration. J Orthop Res. 2019;37:1817‐1826. 10.1002/jor.24324. 31042310 PMC6824921

[72] Fry CS , Johnson DL , Ireland ML , Noehren B . ACL injury reduces satellite cell abundance and promotes fibrogenic cell expansion within skeletal muscle. J Orthop Res. 2017;35:1876‐1885. 10.1002/jor.23502. 27935172 PMC5466509

[73] Kim KK , Sheppard D , Chapman HA . TGF‐β1 signaling and tissue fibrosis. Cold Spring Harb Perspect Biol. 2018;10 10.1101/cshperspect.a022293. PMC5880172 28432134

[74] Hinz B . Formation and function of the myofibroblast during tissue repair. J Invest Dermatol. 2007;127:526‐537. 10.1038/sj.jid.5700613. 17299435

[75] Contreras O , Rebolledo DL , Oyarzún JE , Olguín HC , Brandan E . Connective tissue cells expressing fibro/adipogenic progenitor markers increase under chronic damage: relevance in fibroblast‐myofibroblast differentiation and skeletal muscle fibrosis. Cell Tissue Res. 2016;364:647‐660. 10.1007/s00441-015-2343-0. 26742767

