# Supplementary material for: Intervertebral disc herniation effects on multifidus muscle composition and resident stem cell populations
Source: JOR Spine. 2020 May 6;3(2):e1091. doi: 10.1002/jsp2.1091 (PMC7323461; doi:10.1002/jsp2.1091)
Supplement: Supplementary file 1 — Table S1 Supporting information [file JSP2-3-e1091-s001.docx]

| Gene | Primer Sequences |
| --- | --- |
| Adiponectin | Forward GCAGTCTGTGGTTCTGATTCCATAC  Reverse GCCCTTGAGTCGTGGTTTCC |
| Fatty Acid Binding Protein 4 (FABP4) | Forward ATGGGATGGAAAATCAACCA  Reverse TGCTTGCTAAATCAGGGAAAA |
| CCAAT Enhancer Binding Protein Beta (CEBP) | Forward ATGGGATGGAAAATCAACCA  Reverse TGCTTGCTAAATCAGGGAAAA |
| Leptin | Forward AGGGAGACCGAGCGCTTTC  Reverse TGCATCTCCACACACCAAACC |
| CD36 | Forward GGGAAAGTCACTGCGACATGAT  Reverse ACGTCGGATTCAAATACAGCATAGA |
| Uncoupling Protein 1 (UCP1) | Forward GTGTGCCCAACTGTGCAATG  Reverse CCAGGATCCAAGTCGCAAGA |
| alpha-Smooth Muscle Actin (aSMA) | Forward TGCCTGCATGGGCAAGTGA  Reverse CTGGGCAGCGGAAACGGCA |
| Lipoprotein Lipase (LPL) | Forward TCATTCCCGGAGTAGCAGAGT  Reverse GGCCACAAGTTTTGGCACC |
| Protein tyrosine phosphatase SHP2 (SHP2) | Forward AAAGGGGAGAGCAATGACGG  Reverse CTCCACCAACGTCGTATTTCA |
| TATA-Box Binding Protein (TBP) | Forward TATCGTGGTCACACTTGTTGAG  Reverse ACCATCCTCTGAGACCGTTTT |
| Adipsin | Forward GACACCATCGACCACGACC  Reverse GCCACGTCGCAGAGAGTTC |
| Insulin Receptor Related Receptor (IR) | Forward GCCTGGATATTCGCTCAGAGG  Reverse GCGGATGACTGCTAGGTTGG |
| SRY-Box Transcription Factor 2 (SOX2) | Forward GCTACAGCATGATGCAGGACCA  Reverse TCTGCGAGCTGGTCATGGAGTT |
| Homeobox Transcription Factor Nanog (NANOG) | Forward TCCCGAGAAAAGATTAGTCAGCA  Reverse AGTGGGGCACCTGTTTAACTT |
| Octamer-binding Transcription Factor 4 (OCT4) | Forward CCTGAAGCAGAAGAGGATCACC  Reverse AAAGCGGCAGATGGTCGTTTGG |
| Marker of Proliferation Ki-67 (Ki67) | Forward GGGCCAATCCTGTCGCTTAAT  Reverse GTTATGCGCTTGCGAACCT |
| Myoblast Determination Protein (MYOD) | Forward CGCCATCCGCTATATCGAGG  Reverse CTGTAGTCCATCATGCCGTCG |
| Myogenin (MYOG) | Forward GGGGAAAACTACCTGCCTGTC  Reverse AGGCGCTCGATGTACTGGAT |
| Myosin Heavy Chain (MYHC) | Forward GGGAGACCTAAAATTGGCTCAA  Reverse TTGCAGACCGCTCATTTCAAA |
